# COVID-19 and monkeypox co-infection: A rapid systematic review

**DOI:** 10.3389/fimmu.2022.1094346

**Published:** 2022-12-14

**Authors:** Amr Ehab El-Qushayri, Abdullah Reda, Jaffer Shah

**Affiliations:** ^1^ Faculty of Medicine, Minia University, Minia, Egypt; ^2^ Faculty of Medicine, Al-Azhar University, Cairo, Egypt; ^3^ Department of Public Health, New York State Department of Health, New York, NY, United States

**Keywords:** COVID19, monkeypox, coinfection - disease, SARS- CoV2, infection

## Abstract

In this paper we aimed to study the characteristics, laboratory data and outcomes of monkeypox virus (MPV) and COVID-19 co-infection. On 2^nd^ October 2022, we used the search term “(“monkeypox virus” OR “MPV” OR “monkey pox” OR “monkeypox”) AND (“COVID-19” OR “COVID 19” OR “novel coronavirus” OR “SARS−CoV−2”)” in five databases to collect the relevant articles. We found three male patients, who had sex with men prior to the infection, had multiple comorbid conditions, were diagnosed with PCR, and were admitted to the hospital. The length of hospital stay was 4, 6, and 9 days. On admission, two cases had multiple vesicular lesions on various sites of the body associated with tonsillar inflammation, while the third case had genital ulcers and inguinal lymph node enlargement. All cases were managed in the hospital and recovered well. It might still be too early to establish solid evidence about the exact cause-effect association between SARS-CoV-2 and MPV co-infection and patient’s outcomes because of the current low sample size. Accordingly, future relevant investigations, estimating the risk ratio of this association are needed to formulate definite evidence.

COVID-19 still constitutes a remarkable burden on countries and their healthcare systems even with the remarkable advances in the vaccine industry. COVID-19 patients are liable to various complications, and those with co-morbidities and associated infections are usually the most vulnerable (1, 2). Recently, a multi-country outbreak of monkeypox virus (MPV) occurred and the World Health Organization declared it a Public Health Emergency of International Concern. Accordingly, certain concerns aroused regarding the outcomes of patients co-infected with COVID-19 and MPV.

To collect all the papers that reported COVID-19 and MPV co-infection, we searched in five databases: PubMed (131), Virtual Health Library (122), Google Scholar (75), Web of Science (79), and Scopus (108). On 2^nd^ October 2022, we used the search term “(“monkeypox virus” OR “MPV” OR “monkey pox” OR “monkeypox”) AND (“COVID-19” OR “COVID 19” OR “novel coronavirus” OR “SARS−CoV−2”)” through all the databases and adapted it to fit the search process in each database. The literature search resulted in 3 articles reporting 3 cases of COVID-19 and MPXV co-infection (**Table 1**). All cases were male patients, who had sex with men prior to the infection, had multiple comorbid conditions, were diagnosed with PCR, and were admitted to the hospital. The length of hospital stay was 4, 6, and 9 days. On admission, two cases had multiple vesicular lesions on various sites of the body associated with tonsillar inflammation, while the third case had genital ulcers and inguinal lymph node enlargement. All cases were managed in the hospital and recovered well.

**Table 1:** Characteristics of patients with COVID-19 and MPV co-infection.

Study ID	Country	Age	Sex	Comorbidities	History of traveling prior to MPV	Sex with men prior to MPV infection	COVID-19 vaccination	MPV and COVID-19 diagnosis	Symptoms and signs at admission	Laboratory findings	X-ray findings	Length of hospital	Treatment	Outcome
Knopp-2022 (3)	USA	38	Male	HIV, Herpes and IV drug use	No	Yes	No	PCR	Tonsillar inflammation, dry mucous membranes, vesiculopapular lesions on face, torso, extremities and genitalia	Elevated WBCs	–	9 days	IV fluids + continuation of the HIV treatment of the patient	Recovery and discharged
Nolasco-2022 (4)	Italy	36	Male	Bipolar disorder and syphilis	Yes	Yes	Yes	PCR	Fever, pharyngodynia, fatigue, headache, dotted body, including the palm of the right hand and the perianal region, skin lesions in various stages of progression, small vesicles, reddened haloed pustules, and umbilicated plaques, bilateral tonsillar hypertrophy, hepatosplenomegaly, and an enlarged hypomobile and painful lymph node in the right inguinal region.	Elevated CRP, fibrinogen, prothrombin time, and positive for HIV	Chest X-ray revealed a parenchymal hypodiaphany in the right parailary region.	6 days	Sotrovimab 500 mg IV + dolutegravir, abacavir and lamivudine for HIV	Recovery and discharged
Vives-2022 (5)#	Spain	56	Male	Depression, type 2 diabetes mellitus, and previous hospital admission for COVID-19	No	Yes	–	PCR	Genital ulcer with indurate edges and a fibrin base, foreskin edema, glans erythema, painful bilateral inguinal lymph nodes swelling, and two punctate erythematous lesions on the extremities. After 24h of admission, new blistering, pustular and ulcerated lesions on the extremities, buttocks, chest, and scalp	Elevated WBCs, CRP, and glycemia	–	4 days	A single dose of 2.4 million units of Benzathine penicillin and amoxicillin-clavulanic acid 875/125 mg TID iv	Recovery and discharged

#Preprint paper; HIV, human immunodeficiency virus; IV, intravenous; MPXV, monkeypox virus; PCR, polymerase chain reaction; WBCs, white blood cells; CRP, C-reactive protein.

Our findings indicated that although all the included three cases with the co-infection were hospitalized, none of them developed severe outcomes and were discharged after a good recovery. Accordingly, these findings showed that patients having COVID-19 and MPV co-infection might not develop severe outcomes since all the included patients also had multiple comorbid conditions, which were previously reported as significant risk factors for developing severe COVID-19 outcomes (2). To our knowledge, COVID-19 infection presents usually with fever, cough, fever and smell and tasting disorders. However, the characteristic vesicular and ulcerative lesions in the genital lesions together with the enlargement of lymph nodes in our series are not common features of the disease and suggested co-infection with MPV based upon the manifestations reported in the published literature (6, 7). In our series of patients, elevated white blood cells was noticed in two of three patients, which confirms other comorbid infectious condition rather than COVID-19 that is not associated with WBCs rise in most cases (8). The estimated favorable outcomes are consistent with the findings of our previous report of 4080 MPV patients, where no mortality or intensive care unit admissions were estimated (6). In another report, the estimated case-fatality rate of MPV in non-endemic regions is 0.01% (9). The length of hospital stay was also not long among the included patients, ranging between 4 and 9 days. Besides, supportive management seems to be sufficient for these patients due to the mild course of the disease. Therefore, it can be suggested –until now- that MPV infection, whether alone or in combination with COVID-19 does not induce severe patient’s outcomes. However, it might still be too early to establish solid evidence about the exact cause-effect association between SARS-CoV-2 and MPV co-infection and patient’s outcomes because of the current low sample size. Accordingly, future relevant investigations, estimating the risk ratio of this association are needed to formulate definite evidence.

Another important aspect to consider is the overlapping of the clinical manifestations between the two infections since most MPV patients present with fever which is also common among COVID-19 patients. Although rash might significantly distinguish the two infections, some MPV patients might suffer from atypical undetectable lesions and others might suffer from associated rash-causing illnesses (5), making it difficult to establish a proper differential diagnosis. However, the current MPV outbreak characteristically spreads among men who have sex with men. Accordingly, a history of such sexual contact, together with laboratory-detecting MPV, might provide a sufficient differential diagnosis. Therefore, healthcare physicians should be aware of the best practices to establish a differential diagnosis and have adequate knowledge about the characteristics of MPV. Awareness campaigns are essential in this context since the disease, despite re-emerging, is novel in some countries and hard to identify by many healthcare providers (5).

Early detection of cases might also another factor responsible for the reduced severity of co-infection. Therefore, establishing an early proper diagnosis and detection of infections is vital in curbing the spread of these diseases. Moreover, inaugurating national surveillance programs targeting the most impacted populations and populations at risk of catching the infection is also important. Finally, one patient received COVID-19 vaccination, which might also be protective and responsible for the mild outcomes. However, one patient did not receive the vaccine and still had favorable outcomes, and no information was available for the other, indicating the need for future relevant investigations.

## Author contributions

AE-Q was responsible for the idea and the study design. All authors extracted the data and shared in the writing of the full text and approval of final version before submission.
